# Association between incorrect posture and curve types in adolescent idiopathic scoliosis: a large-sample, cross-sectional study in China

**DOI:** 10.3389/fpubh.2026.1785027

**Published:** 2026-06-12

**Authors:** Xi Yu, Rui Zhang, Xiaosheng Chen, Zhixiang Zhu, Yuchen Zheng, Weijun Wang, Fei Yu, WenYu Zhou, Lei Yang, Qian Liang

**Affiliations:** 1 Department of Spine Surgery, The Shenzhen Second People’s Hospital, The First Affiliated Hospital of Shenzhen University, Shenzhen, China; 2 Shenzhen Youth Spine Health Center, Shenzhen, China; 3 Medical Innovation Technology Transformation Center of Shenzhen Second People’s Hospital, Shenzhen, China

**Keywords:** adolescent, curve type, incorrect posture, scoliosis, screening

## Abstract

**Background:**

Screening for adolescent idiopathic scoliosis (AIS) has progressed considerably in recent years. However, specific physical indicators for accurately determining spinal curve types are lacking. This study aimed to explore the association between incorrect posture and curve types in AIS.

**Methods:**

We analyzed data from 10,453 students who underwent full-spine evaluation in the outpatient department between 2022 and 2023. Assessments included postural observations, Adam’s Forward Bending Test, angle of trunk rotation (ATR), and full-spine radiographic imaging. Correlation and logistic regression (LR) models were used to analyze the associations among incorrect posture, curve magnitude, and curve type.

**Results:**

Incorrect postures, particularly the presence of a rib hump, were significantly correlated with the curve magnitude in AIS. Univariate LR analysis showed that female, age, Risser sign, and incorrect postures were identified as associated factors for the curve type. Multivariate LR analysis revealed that right scapular tilt [odds ratio (OR): 1.553, 95% confidence interval (CI): 1.192–2.025, *p* = 0.001], right rib hump (OR: 1.715, 95% CI: 1.451–2.028, *p* < 0.001), and right thoracic ATR ≥ 5° (OR: 1.769, 95% CI: 1.484–2.109, *p* < 0.001) were associated with the thoracic curve; right pelvic tilt (OR: 1.411, 95% CI: 1.253–1.588, *p* < 0.001) and right rib hump (OR: 1.452, 95% CI: 1.215–1.736, *p* < 0.001) were associated with the thoracolumbar curve; and left pelvic tilt (OR: 1.266, 95% CI: 1.085–1.477, *p* = 0.003), right pelvic tilt (OR: 1.321, 95% CI: 1.175–1.485, *p* = 0.003), and left lumbar eminence (OR: 1.229, 95% CI: 1.033–1.462, *p* = 0.020) were associated with the lumbar curve.

**Conclusion:**

Incorrect posture and ATR, especially right rib hump, were significantly associated with curve magnitude and curve type in AIS. These indicators can provide an objective reference for triaging children referred from school-based screening for AIS, facilitating a more targeted and efficient identification of at-risk individuals.

## 1 Introduction

Adolescent idiopathic scoliosis (AIS) is the most prevalent three-dimensional spinal deformity that occurs during puberty and has an unclear etiology (1). Globally, the incidence of AIS ranges from 0.47 to 5.20%, with a higher prevalence in females than in males (2). In the early stages, affected individuals often present with mild or no symptoms, contributing to frequent underdiagnosis (3). If left untreated, AIS may progress to severe spinal deformity, resulting in trunk asymmetry, cardiopulmonary compromise, psychological distress, and a significantly diminished quality of life (4, 5). Approximately 10% of patients with AIS exhibit a progressive curve (6). Furthermore, patients with advanced AIS who require surgical treatment often experience not only physical discomfort and appearance anxiety caused by the physical deformity but also significant psychological stress over the long term, which can affect their emotional development during adolescence (7). Additionally, the high expenses associated with spinal orthopedic surgeries and subsequent rehabilitation treatments often impose a considerable financial burden on families (8). Studies have shown that repeated full-spine radiographic examinations may increase the risk of breast cancer in female patients with AIS, and the cumulative radiation dose exhibits a positive correlation with the risk of hematologic malignancies (9, 10). Therefore, early screening and diagnosis based on physical signs are particularly important in adolescents.

With growing social awareness of the spinal health of teenagers, early detection and intervention of AIS have become key concerns in both clinical and public health domains. Since 2013, our team at the Shenzhen Youth Spine Health Center in China has conducted a scoliosis screening program for primary and secondary school students in Shenzhen, systematically testing student populations at risk of developing a spinal deformity to provide a scientific basis for early intervention (11). According to our research, among the 1,793,787 primary and secondary school students screened in the Shenzhen school-based scoliosis screening program, 79.92% exhibited postural abnormalities, including shoulder asymmetry, scapular tilt, and pelvic tilt (12). In particular, shoulder tilt and asymmetry on the left and right sides were more pronounced (13). Our preliminary findings revealed a significant correlation between the right rib hump and the curve magnitude in AIS (14). Other studies have demonstrated significant associations among pelvic morphology, trunk posture, and Cobb angle (15). Overall, previous studies and our own initial data suggest a correlation between postural abnormalities and the magnitude of the Cobb angle. In addition, curve type plays a crucial role in the treatment of AIS. Therefore, early detection of incorrect posture could aid in predicting the curve magnitude and type in AIS, ultimately contributing to improved diagnosis and treatment outcomes in school-based scoliosis screening. However, there is a lack of systematic research exploring the relationship between incorrect postures and different curve types (e.g., thoracic, thoracolumbar, and lumbar curves). Whether an incorrect posture is associated with a specific curve type and can serve as a reliable predictor remains unclear.

Therefore, in this study, we enrolled 10,453 patients with suspected spinal curvature from the Chinese School-Based Scoliosis Screening Program (CSSSP) and performed full-spine radiographic examinations in the outpatient department between 2022 and 2023. To our knowledge, this is the first study to investigate the correlation between external postural signs and different curve types in a large sample. Our results aim to help identify the indicators of curve magnitude and type, providing a scientific foundation for targeted triage and management of children identified during school-based screening.

## 2 Materials and methods

### 2.1 CSSSP

The CSSSP is part of a national public health initiative designed to identify spinal health issues in children and adolescents, covering primary, junior high, and senior high school students in the Shenzhen area. The screening initiative is implemented by the Shenzhen Youth Spine Health Center (SYSHC), affiliated with the Shenzhen Second People’s Hospital, in strict compliance with the Chinese National Standards (GB/T 16133–2014). In this study, the screening team consisted of professional rehabilitation therapists from the SYSHC who adopted a standardized screening process. This included visual observation, Adam’s Forward Bend Test (FBT), and measurement of the angle of trunk rotation (ATR) using a scoliometer (16). Students identified as having suspected AIS during the screening process were referred to the hospital’s outpatient department for a standing-position full-spine radiography examination to determine the severity and classification of the spinal curvature (17).

### 2.2 Data collection and measurements

This study included students who were identified as having suspected scoliosis during campus-based screening and subsequently underwent follow-up examinations at the hospital’s outpatient department between 2022 and 2023. These examinations included physical examination of the body surface and full-spine radiography. Posture assessment includes multiple parameters, such as observing the shoulder height difference, scapular tilt, pelvic tilt, rib hump, and lumbar concavity in the upright position. Additionally, in Adam’s FBT, a scoliometer was used to measure the rotation angles of the thoracic, thoracolumbar, and lumbar spine. Detailed measurement procedures are provided in Supplementary File 1 (operational definitions of postural signs). To ensure the reliability of the assessment results, physical examinations for each student were performed by two rigorously trained and qualified independent therapists. In cases where the assessment results differed between the two therapists, a third senior therapist made the final determination. The specific methods for conducting physical measurements have been detailed in our previously published studies (18, 19). Full-spine radiography was recommended for students when the ATR was ≥ 5° (20). This study was approved by the Ethics Committee of Shenzhen Second People’s Hospital, and written informed consent was obtained from all participants and their guardians.

### 2.3 Definition of the curve type

The Cobb angle was measured using full-spine radiographs. Patients with a Cobb angle of > 10° were classified according to the curve type, according to the classification standard of the Scoliosis Research Society. The categorization is determined by the location of the apical vertebra in the spinal curve. Specifically, (1) thoracic curve: the apical vertebra is located between T2 and T11; (2) thoracolumbar curve: the upper vertebra is located in the thoracic region, the lower vertebra is located in the lumbar region, and the apical vertebra is located between T12 and L1; and (3) lumbar curve: the spinal curve is located in the lumbar region, with the apical vertebra located between L2 and L5. For patients with multiple spinal curves, only the major (primary) curve was included in the analysis.

### 2.4 Statistical analysis

All data were analyzed using SPSS 20.0 and the R language. Descriptive statistics were used to summarize the demographic characteristics, prevalence of incorrect postures, and ATR measurements. The Mann–Whitney U test was used to compare two groups (e.g., sex, hump back, and lumbar lordosis), whereas the Kruskal–Wallis test was employed for comparing three or more independent groups (e.g., age, incorrect postures, and ATR) stratified by major curve magnitude (Cobb angle: < 10°, 10–19°, 20–39°, and ≥ 40°) and curve type. Univariate logistic regression (LR) models were used to preliminarily explore the correlation between screening items (such as sex, age, incorrect postures, and ATR) and curve types. Multivariate LR analysis was performed to determine the independent effects of each indicator of incorrect posture on the curve. Odds ratios (ORs) and 95% confidence intervals (CIs) were obtained from the LR models. For multiple chi-square tests across categorical variables, the Benjamini-Hochberg false discovery rate (FDR) method was applied to adjust *p*-values. A *p*-value of < 0.05 was considered statistically significant.

## 3 Results

### 3.1 Demographic characteristics of the study population

A total of 10,453 students aged 6–18 years were included in this study, with the majority concentrated in the 11–15-year age group. The cohort consisted of 3,542 males and 6,911 females, with most students enrolled in primary and junior high schools. The number of students with Risser signs 0 and 4 was the highest. Based on the distribution characteristics of the curve types, the patients were divided into a normal group (Cobb angle < 10°; *n* = 3,218), thoracic curve group (*n* = 2,968), thoracolumbar curve group (*n* = 2,372), and lumbar curve group (*n* = 1,895; Table 1).

**Table 1:** Demographic characteristics of the included population.

Variables	Frequency (N)	Percent(%)
Total	10,453	100.00
Gender		
Boys	3,542	33.89
Girls	6,911	66.11
Age (year)		
6	121	1.16
7	286	2.74
8	397	3.8
9	529	5.06
10	936	8.95
11	1,256	12.02
12	1,686	16.13
13	1710	16.36
14	1,412	13.51
15	1,100	10.52
16	810	7.75
17	199	1.9
18	11	0.11
School category		
Primary school	4,189	40.07
Junior high school	4,499	43.04
Senior high school	1765	16.89
Grade		
Grade 1	247	2.36
Grade 2	349	3.34
Grade 3	437	4.18
Grade4	694	6.64
Grade5	1,094	10.47
Grade6	1,368	13.09
Grade7	1733	16.58
Grade8	1,635	15.64
Grade9	1,131	10.82
Grade10	1,043	9.98
Grade11	707	6.76
Grade12	15	0.14
Risser sign		
0	3,247	31.06
1	548	5.24
2	846	8.09
3	1758	16.82
4	3,168	30.31
5	886	8.48
Curve type		
Normal	3,218	30.79
Thoracic curve	2,968	28.39
Thoracolumbar curve	2,372	22.69
Lumbar curve	1895	18.13

N, number; Normal, Cobb angle < 10°.

### 3.2 Association of incorrect posture with the curve magnitude

Next, we explored the relationship between incorrect posture and curve magnitude. As presented in Table 2, the proportion of female patients with AIS was higher than that of male patients across all curve magnitude groups (Cobb angle 10–19°: 67.5% vs. 32.5%; 20–39°: 83.59% vs. 16.41%; ≥ 40°: 79.31% vs. 20.69%, respectively; *χ*^2^ = 512.801, *p* < 0.001). Most AIS patients were aged between 13 and 15 years (*χ*^2^ = 221.563, *p* < 0.001), and the proportion of patients with a Risser sign 4 was the highest (*χ*^2^ = 363.692, *p* < 0.001). Scapular tilt (*χ*^2^ = 87.999, *p* < 0.001), lumbar concavity (*χ*^2^ = 186.838, *p* < 0.001), pelvic tilt (*χ*^2^ = 250.796, *p* < 0.001), rib hump (*χ*^2^ = 479.152, *p* < 0.001), and thoracic ATR (*χ*^2^ = 501.819, *p* < 0.001) demonstrated significant differences among the curve magnitude groups. The prevalence of right shoulder height, right scapular tilt, lumbar concavity, pelvic tilt, rib hump, and right thoracic ATR significantly increased as the Cobb angle increased. Notably, in the > 40° group, 72.41% of patients exhibited the right rib hump and 67.24% had a right thoracic ATR of ≥ 5°. Overall, sex, Risser sign, shoulder height difference, scapular tilt, lumbar concavity, pelvic tilt, rib hump, and thoracic ATR are significantly associated with the curve magnitude.

**Table 2:** Incorrect postures of participants stratified by Cobb angle distribution.

Variables	Distribution of Cobb angle *n* (%)				Z^*^ /χ2^#^	*p*
	< 10°	10–19°	20–39°	≥ 40°		
Total	3,218	5,230	1889	116		
Gender						
Boys	1,508 (46.86%)	1700 (32.50%)	310 (16.41%)	24 (20.69%)	512.801	**< 0.001**
Girls	1710 (53.14%)	3,530 (67.50%)	1,579 (83.59%)	92 (79.31%)		
Age (year)						
6–10	946 (29.40%)	1,064 (20.34%)	249 (13.18%)	10 (8.62%)	221.563	**< 0.001**
11–15	1980 (61.53%)	3,617 (69.16%)	1,470 (77.82%)	97 (83.62%)		
16–18	292 (9.07%)	549 (10.50%)	170 (9.00%)	9 (7.76%)		
Risser sign						
0	1,356 (42.14%)	1,512 (28.91%)	363 (19.22%)	16 (13.79%)	363.692	**< 0.001**
1	130 (4.04%)	306 (5.85%)	109 (5.77%)	3 (2.59%)		
2	233 (7.24%)	447 (8.55%)	155 (8.21%)	11 (9.48%)		
3	478 (14.85%)	858 (16.41%)	396 (20.96%)	26 (22.41%)		
4	778 (24.18%)	1,635 (31.26%)	706 (37.37%)	49 (42.25%)		
5	243 (7.55%)	472 (9.02%)	160 (8.47%)	11 (9.48%)		
Coronal plane						
Shoulder-height difference						
Normal	483 (15.01%)	655 (12.52%)	171 (9.05%)	8 (6.90%)	47.658	**< 0.001**
Left shoulder height	1,331 (41.36%)	2,279 (43.58%)	814 (43.09%)	45 (38.79%)		
Right shoulder height	1,404 (43.63%)	2,296 (43.90%)	904 (47.86%)	63 (54.31%)		
Scapula tilt						
Normal	549 (17.06%)	687 (13.14%)	168 (8.89%)	6 (5.17%)	87.999	**< 0.001**
Left tilt	1,357 (42.17%)	2,293 (43.84%)	818 (43.30%)	43 (37.07%)		
Right tilt	1,312 (40.77%)	2,250 (43.02%)	903 (47.81%)	67 (57.76%)		
Lumbar concave						
Normal	1,376 (42.76%)	1857 (35.51%)	484 (25.62%)	14 (12.07%)	186.838	**< 0.001**
Left concave	751 (23.34%)	1,333 (25.48%)	596 (31.55%)	46 (39.66%)		
Right concave	1,091 (33.90%)	2040 (39.01%)	809 (42.83%)	56 (48.27%)		
Pelvic tilt						
Normal	2,472 (76.82%)	3,696 (70.67%)	1,107 (58.60%)	52 (44.83%)	250.796	**< 0.001**
Left tilt	305 (9.48%)	520 (9.94%)	225 (11.91%)	20 (17.24%)		
Right tilt	441 (13.70%)	1,014 (19.39%)	557 (29.49%)	44 (37.93%)		
Sagittal plane						
Flat back						
Normal	3,209 (99.72%)	5,211 (99.64%)	1875 (99.26%)	115 (99.14%)	7.247	0.084
Abnormal	9 (0.28%)	19 (0.36%)	14 (0.74%)	1 (0.86%)		
Hump back						
Normal	3,173 (98.60%)	5,174 (98.93%)	1880 (99.52%)	116 (100%)	10.968	**0.017**
Abnormal	45 (1.4%)	56 (1.07%)	9 (0.48%)	0 (0%)		
Lumbar lordosis						
Normal	3,216 (99.94%)	5,227 (99.94%)	1889 (100%)	116 (100%)	1.197	0.754
Abnormal	2 (0.06%)	3 (0.06%)	0 (0%)	0 (0%)		
Lumbar kyphosis						
Normal	3,196 (99.32%)	5,202 (99.46%)	1884 (99.74%)	116 (100%)	4.615	0.245
Abnormal	22 (0.68%)	28 (0.54%)	5 (0.26%)	0 (0%)		
Adam’s test						
Rib hump						
Normal	1872 (58.17%)	2,761 (52.79%)	750 (39.70%)	17 (14.66%)	479.152	**< 0.001**
Left eminence	551 (17.12%)	659 (12.60%)	159 (8.42%)	15 (12.93%)		
Right eminence	795 (24.70%)	1810 (34.61%)	980 (51.88%)	84 (72.41%)		
Angle of thoracic rotation						
Normal (ATR 0–4°)	2,252 (69.98%)	3,369 (64.42%)	917 (48.54%)	26 (22.42%)	501.819	**< 0.001**
Left (ATR ≥ 5°)	347 (10.78%)	441 (8.43%)	115 (6.09%)	12 (10.34%)		
Right (ATR ≥ 5°)	619 (19.24%)	1,420 (27.15%)	857 (45.37%)	78 (67.24%)		
Thoracolumbar eminence						
Normal	2,841 (88.28%)	4,610 (88.15%)	1,684 (89.15%)	100 (86.21%)	4.838	0.600
Left eminence	146 (4.54%)	233 (4.45%)	82 (4.34%)	9 (7.76%)		
Right eminence	231 (7.18%)	387 (7.40%)	123 (6.51%)	7 (6.03%)		
Angle of thoracolumbar rotation						
Normal (ATR 0–4°)	2,891 (89.84%)	4,690 (89.67%)	1709 (90.47%)	100 (86.21%)	6.086	0.469
Left (ATR ≥ 5°)	126 (3.92%)	201 (3.85%)	72 (3.81%)	9 (7.76%)		
Right (ATR ≥ 5°)	201 (6.24%)	339 (6.48%)	108 (5.72%)	7 (6.03%)		
Lumbar eminence						
Normal	1,368 (42.51%)	2,222 (42.49%)	707 (37.43%)	42 (36.21%)	124.741	**< 0.001**
Left eminence	1,225 (38.07%)	2069 (39.56%)	963 (50.98%)	66 (56.89%)		
Right eminence	625 (19.42%)	939 (17.95%)	219 (11.59%)	8 (6.9%)		
Angle of lumbar rotation						
Normal (ATR 0–4°)	1720 (53.45%)	2,860 (54.68%)	956 (50.61%)	47 (40.51%)	80.727	**< 0.001**
Left (ATR ≥ 5°)	1,027 (31.91%)	1,637 (31.30%)	750 (39.70%)	61 (52.59%)		
Right (ATR ≥ 5°)	471 (14.64%)	733 (14.02%)	183 (9.69%)	8 (6.9%)		

*n*, number; ATR, angle of trunk rotation. * Mann–Whitney U test; ^#^ Kruskal-Wallis test. The bold numbers of p value represent the significant differences.

### 3.3 Association of incorrect posture with the curve type

We further analyzed the correlation between the curve type and the incorrect posture. As presented in Table 3, female patients had a higher prevalence of AIS than male patients across all curve type groups (*χ*^2^ = 351.163, *p* < 0.001), and patients were aged between 11 and 15 years (*χ*^2^ = 194.4, *p* < 0.001). In the thoracic, thoracolumbar, and lumbar curve groups, the incidences of left shoulder height (44.68, 43.04, and 41.74%, respectively), right shoulder height (47.07, 42.71, and 45.01%, respectively), left scapular tilt (44.68, 43.30, and 42.27%, respectively), and right scapular tilt (46.66, 41.99, and 44.27%, respectively) were all higher than those of normal shoulder height (8.25, 14.25, and 13.25%, respectively; *χ*^2^ = 76.284, *p* < 0.001) and normal scapular (8.66, 14.71, and 13.46%, respectively; *χ*^2^ = 100.911, *p* < 0.001). Interestingly, in the thoracolumbar and lumbar curve groups, the incidences of right lumbar concavity were 45.28 and 47.65%, respectively, which were higher than those of normal (29.68 and 31.03%, respectively) or left lumbar concavity (25.04 and 21.32%, respectively; *χ*^2^ = 288.786, *p* < 0.001). Furthermore, the proportion of right rib hump in the thoracic curve group (51.11%) was higher than that in the normal and left rib hump groups (35.78 and 10.11%, respectively; *χ*^2^ = 714.969, *p* < 0.001). Correspondingly, 47.54% of patients had a right thoracic ATR of ≥ 5° in the thoracic curve group, which was significantly higher than those with a left thoracic ATR (7.51%; *χ*^2^ = 775.77, *p* < 0.001). Moreover, 50.92% of patients had left lumbar eminence in the lumbar curve group (*χ*^2^ = 206.779, *p* < 0.001). In conclusion, sex, age, shoulder height difference, scapular tilt, lumbar concavity, rib hump, and right thoracic ATR ≥ 5° are significantly associated with the curve type.

**Table 3:** Incorrect postures of participants stratified by curve types distribution.

Variables	Curve type *n* (%)				Z^*^ /χ2^#^	*P*
	Normal	TC	TLC	LC		
Total	3,218	2,968	2,372	1895		
Gender						
Boys	1,508 (46.86%)	858 (28.91%)	662 (27.91%)	514 (27.12%)	351.163	**< 0.001**
Girls	1710 (53.14%)	2,110 (71.09%)	1710 (72.09%)	1,381 (72.88%)		
Age (year)						
6–10	946 (29.40%)	584 (19.68%)	479 (20.19%)	260 (13.72%)	194.4	**< 0.001**
11–15	1980 (61.53%)	2099 (70.72%)	1,656 (69.82%)	1,429 (75.41%)		
16–18	292 (9.07%)	285 (9.60%)	237 (9.99%)	206 (10.87%)		
Risser sign						
0	1,356 (42.14%)	769 (25.91%)	684 (28.84%)	438 (23.11%)	304.322	**< 0.001**
1	130 (4.04%)	172 (5.80%)	140 (5.90%)	106 (5.59%)		
2	233 (7.24%)	275 (9.27%)	185 (7.80%)	153 (8.07%)		
3	478 (14.85%)	540 (18.19%)	414 (17.45%)	326 (17.21%)		
4	778 (24.18%)	960 (32.35%)	731 (30.82%)	699 (36.89%)		
5	243 (7.55%)	252 (8.48%)	218 (9.19%)	173 (9.13%)		
Coronal plane						
Shoulder-height difference						
Normal	483 (15.01%)	245 (8.25%)	338 (14.25%)	251 (13.25%)	76.284	**< 0.001**
Left shoulder height	1,331 (41.36%)	1,326 (44.68%)	1,021 (43.04%)	791 (41.74%)		
Right shoulder height	1,404 (43.63%)	1,397 (47.07%)	1,013 (42.71%)	853 (45.01%)		
Scapula tilt						
Normal	549 (17.06%)	257 (8.66%)	349 (14.71%)	255 (13.46%)	100.911	**< 0.001**
Left tilt	1,357 (42.17%)	1,326 (44.68%)	1,027 (43.30%)	801 (42.27%)		
Right tilt	1,312 (40.77%)	1,385 (46.66%)	996 (41.99%)	839 (44.27%)		
Lumbar concave						
Normal	1,376 (42.76%)	1,063 (35.82%)	704 (29.68%)	588 (31.03%)	288.786	**< 0.001**
Left concave	751 (23.34%)	977 (32.92%)	594 (25.04%)	404 (21.32%)		
Right concave	1,091 (33.90%)	928 (31.27%)	1,074 (45.28%)	903 (47.65%)		
Pelvic tilt						
Normal	2,472 (76.82%)	2,175 (73.28%)	1,488 (62.73%)	1,192 (62.90%)	259.958	**< 0.001**
Left tilt	305 (9.48%)	317 (10.68%)	282 (11.89%)	166 (8.76%)		
Right tilt	441 (13.70%)	476 (16.04%)	602 (25.38%)	537 (28.34%)		
Sagittal plane						
Flat back						
Normal	3,209 (99.72%)	2,948 (99.33%)	2,363 (99.62%)	1890 (99.74%)	7.42	0.077
Abnormal	9 (0.28%)	20 (0.67%)	9 (0.38%)	5 (0.26%)		
Hump back						
Normal	3,173 (98.60%)	2,949 (99.36%)	2,343 (98.78%)	1878 (99.10%)	9.642	**0.031**
Abnormal	45 (1.40%)	19 (0.64%)	29 (1.22%)	17 (0.90%)		
Lumbar lordosis						
Normal	3,216 (99.94%)	2,967 (99.97%)	2,370 (99.92%)	1895 (100%)	1.829	0.609
Abnormal	2 (0.06%)	1 (0.03%)	2 (0.08%)	0 (0%)		
Lumbar kyphosis						
Normal	3,196 (99.32%)	2,955 (99.56%)	2,364 (99.66%)	1883 (99.37%)	3.998	0.300
Abnormal	22 (0.68%)	13 (0.44%)	8 (0.34%)	12 (0.63%)		
Adam’s test						
Rib hump						
Normal	1872 (58.17%)	1,062 (35.78%)	1,365 (57.55%)	1,101 (58.10%)	714.969	**< 0.001**
Left eminence	551 (17.12%)	300 (10.11%)	333 (14.04%)	200 (10.55%)		
Right eminence	795 (24.71%)	1,606 (51.11%)	674 (28.41%)	594 (31.35%)		
Angle of thoracic rotation						
Normal (ATR 0–4°)	2,252 (69.98%)	1,334 (44.95%)	1,647 (69.44%)	1,331 (70.24%)	775.77	**< 0.001**
Left (ATR ≥ 5°)	347 (10.78%)	223 (7.51%)	215 (9.06%)	130 (6.86%)		
Right (ATR ≥ 5°)	619 (19.24%)	1,411 (47.54%)	510 (21.50%)	434 (22.90%)		
Thoracolumbar eminence						
Normal	2,841 (88.28%)	2,685 (90.46%)	2013 (84.87%)	1,696 (89.50%)	59.947	**< 0.001**
Left eminence	146 (4.54%)	86 (2.90%)	164 (6.91%)	74 (3.90%)		
Right eminence	231 (7.18%)	197 (6.64%)	195 (8.22%)	125 (6.60%)		
Angle of thoracolumbar rotation						
Normal (ATR 0–4°)	2,891 (89.84%)	2,723 (91.75%)	2052 (86.51%)	1724 (90.98%)	61.019	**< 0.001**
Left (ATR ≥ 5°)	126 (3.91%)	71 (2.39%)	147 (6.20%)	64 (3.38%)		
Right (ATR ≥ 5°)	201 (6.25%)	174 (5.86%)	173 (7.29%)	107 (5.64%)		
Lumbar eminence						
Normal	1,368 (42.51%)	1,474 (49.66%)	896 (37.77%)	601 (31.72%)	206.779	**< 0.001**
Left eminence	1,225 (38.07%)	1,104 (37.20%)	1,029 (43.39%)	965 (50.92%)		
Right eminence	625 (19.42%)	390 (13.14%)	447 (18.84%)	329 (17.36%)		
Angle of lumbar rotation						
Normal (ATR 0–4°)	1720 (53.45%)	1856 (62.53%)	1,159 (48.86%)	848 (44.75%)	194.641	**< 0.001**
Left (ATR ≥ 5°)	1,027 (31.91%)	815 (27.46%)	845 (35.62%)	788 (41.58%)		
Right (ATR ≥ 5°)	471 (14.64%)	297 (10.01%)	368 (15.51%)	259 (13.67%)		

*n*, number; ATR, angle of trunk rotation. Normal, Cobb angle < 10°. TC, Thoracic curve; TLC, Thoracolumbar curve; LC, Lumbar curve. * Mann–Whitney U test; ^#^Kruskal-Wallis test. The bold numbers of P value represent the significant differences.

### 3.4 Association between influential factors and curve types: univariate and multivariate analyses

Univariate and multivariate regression analyses were conducted to identify the associated factors associated with different curve types in AIS.

#### 3.4.1 Analysis of related factors for the thoracic curve

As presented in Table 4, single-factor LR analysis indicated that female sex, age, Risser sign, shoulder height difference, scapular tilt, lumbar concavity, flat back, hump back, rib hump, thoracic ATR, right thoracolumbar eminence, right thoracolumbar ATR, and left lumbar eminence were significantly associated with the thoracic curve (*p* < 0.05). Multivariate analysis further revealed that female sex (OR: 1.744, 95% CI: 1.588–1.915, *p* < 0.001), Risser sign 4 (OR: 1.641, 95% CI: 1.395–1.931, *p* < 0.001), right scapular tilt (OR: 1.553, 95% CI: 1.192–2.025, *p* = 0.001), left lumbar concavity (OR: 1.141, 95% CI: 1.017–1.279, *p* = 0.024), and right pelvic tilt (OR: 1.260, 95% CI: 1.125–1.411, *p* < 0.001) were independent associated factors for the thoracic curve. In addition, in Adam’s FBT, right rib hump (OR: 1.715, 95% CI: 1.451–2.028, *p* < 0.001) and right thoracic ATR ≥ 5° (OR: 1.769, 95% CI: 1.484–2.109, *p* < 0.001) were identified as associated factors for the thoracic curve. However, left rib hump (OR: 0.620, 95% CI: 0.5–0.77, *p* < 0.001), right lumbar eminence (OR: 0.689, 95% CI: 0.546–0.87, *p* = 0.002), and left lumbar ATR (OR: 0.842, 95% CI: 0.715–0.991, *p* = 0.039) were negatively associated with the thoracic curve. In conclusion, female sex, Risser sign, right scapular tilt, right pelvic tilt, right rib hump, and right thoracic ATR ≥ 5° are associated with the thoracic curve.

**Table 4:** Association between incorrect posture and thoracic curve among adolescents.

Variable	Univariate LR			Multivariate LR		
	OR	95% CI	*p*	OR	95% CI	*p*
Gender						
Boys	1.000			1.000		
Girls	2.169	1.991–2.364	**< 0.001**	1.744	1.588–1.915	**< 0.001**
Age (year)						
<10	1.000			1.000		
10–15	1.706	1.545–1.884	**< 0.001**	1.132	0.973–1.315	0.108
>15	1.429	1.227–1.664	**< 0.001**	1.073	0.831–1.386	0.591
School category						
Primary school	1.000			1.000		
Junior high school	1.405	1.29–1.531	**< 0.001**	0.903	0.794–1.028	0.125
Senior high school	1.194	1.066–1.337	**0.002**	0.801	0.65–0.986	**0.036**
Risser sign						
0	1.000			1.000		
1	1.742	1.45–2.093	**< 0.001**	1.349	1.095–1.662	**0.005**
2	1.828	1.567–2.132	**< 0.001**	1.412	1.172–1.7	**< 0.001**
3	1.825	1.62–2.056	**< 0.001**	1.452	1.234–1.709	**< 0.001**
4	1.934	1.747–2.14	**< 0.001**	1.641	1.395–1.931	**< 0.001**
5	1.575	1.353–1.834	**< 0.001**	1.593	1.261–2.012	**< 0.001**
Coronal plane						
Shoulder-height difference						
Normal	1.000			1.000		
Left height	1.422	1.249–1.618	**< 0.001**	1.171	0.889–1.543	0.262
Right height	1.650	1.452–1.876	**< 0.001**	0.784	0.599–1.027	0.077
Scapula tilt						
Normal	1.000			1.000		
Left tilt	1.493	1.315–1.694	**< 0.001**	1.056	0.805–1.385	0.692
Right tilt	1.826	1.609–2.072	**< 0.001**	1.553	1.192–2.025	**0.001**
Lumbar concave						
Normal	1.000			1.000		
Left concave	1.414	1.279–1.563	**< 0.001**	1.141	1.017–1.279	**0.024**
Right concave	1.228	1.121–1.345	**< 0.001**	1.026	0.923–1.14	0.638
Pelvic tilt						
Normal	1.000			1.000		
Left tilt	1.053	0.925–1.2	0.434	1.033	0.892–1.197	0.665
Right tilt	1.366	1.238–1.507	**< 0.001**	1.260	1.125–1.411	**< 0.001**
Sagittal plane						
Flat back						
Normal	1.000			1.000		
Abnormal	3.193	1.664–6.129	**< 0.001**	2.175	1.103–4.291	**0.025**
Hump back						
Normal	1.000			1.000		
Abnormal	0.613	0.408–0.920	**0.018**	0.787	0.512–1.21	0.276
Adam’s test						
Rib hump						
Normal	1.000			1.000		
Left eminence	0.777	0.683–0.884	**< 0.001**	0.620	0.5–0.77	**< 0.001**
Right eminence	2.605	2.390–2.840	**< 0.001**	1.715	1.451–2.028	**< 0.001**
Angle of thoracic rotation						
Normal (ATR 0–4°)	1.000			1.000		
Left (ATR ≥ 5°)	0.779	0.671–0.906	**0.001**	1.280	0.993–1.65	0.057
Right (ATR ≥ 5°)	2.788	2.550–3.048	**< 0.001**	1.769	1.484–2.109	**< 0.001**
Thoracolumbar eminence						
Normal	1.000			1.000		
Left eminence	0.890	0.736–1.075	0.225	1.390	0.811–2.382	0.230
Right eminence	0.682	0.583–0.798	**< 0.001**	0.874	0.547–1.397	0.575
Angle of thoracolumbar rotation						
Normal (ATR 0–4°)	1.000			1.000		
Left (ATR ≥ 5°)	0.903	0.737–1.105	0.320	0.986	0.557–1.748	0.963
Right (ATR ≥ 5°)	0.703	0.595–0.830	**< 0.001**	1.129	0.689–1.849	0.631
Lumbar eminence						
Normal	1.000			1.000		
Left eminence	1.171	1.075–1.274	**< 0.001**	1.884	1.599–2.221	**< 0.001**
Right eminence	0.666	0.593–0.748	**< 0.001**	0.689	0.546–0.87	**0.002**
Angle of lumbar rotation						
Normal (ATR 0–4°)	1.000			1.000		
Left (ATR ≥ 5°)	1.052	0.966–1.146	0.245	0.842	0.715–0.991	**0.039**
Right (ATR ≥ 5°)	0.641	0.566–0.725	**< 0.001**	1.205	0.935–1.553	0.150

OR, odds ratio; CI, confidence interval; ATR, angle of trunk rotation. The bold numbers of *p* value represent the significant differences.

We used ROC curves and AUC values to compare the predictive performance of ATR for thoracic curve (Supplementary Figure S1). ROC curve analysis revealed that a right thoracic ATR ≥ 5° was the strongest predictor of thoracic curve. The corresponding AUC values are presented in Supplementary Table S1.

#### 3.4.2 Analysis of related factors for the thoracolumbar curve

In the single-factor LR analysis, female sex, age, Risser sign, right shoulder height, right scapular tilt, lumbar concavity, right pelvic tilt, rib hump, thoracic ATR ≥ 5°, thoracolumbar eminence, thoracolumbar ATR ≥ 5°, left lumbar eminence, and left lumbar ATR ≥ 5° were significantly associated with the thoracolumbar curve (*p* < 0.05). After adjusting for multiple factors in the LR analysis, female sex (OR: 1.680, 95% CI: 1.511–1.869, *p* < 0.001), age >10 years, right scapular tilt (OR: 1.436, 95% CI: 1.072–1.924, *p* = 0.015), right lumbar concavity (OR: 1.137, 95% CI: 1.013–1.277, *p* = 0.029), right pelvic tilt (OR: 1.411, 95% CI: 1.253–1.588, *p* < 0.001), right rib hump (OR: 1.452, 95% CI: 1.215–1.736, *p* < 0.001), and left lumbar eminence (OR: 1.762, 95% CI: 1.486–2.089, *p* < 0.001) remained significant. Interestingly, thoracolumbar ATR ≥ 5° was no longer significant in the multivariate analysis. Furthermore, the left rib hump (OR: 0.606, 95% CI: 0.473–0.777, *p* < 0.001) and right shoulder height (OR: 0.704, 95% CI: 0.525–0.944, *p* = 0.019) were negatively associated with the thoracolumbar curve. These results indicate that the right scapular tilt, right lumbar concavity, right pelvic tilt, right rib hump, and left lumbar eminence are independent associated factors for the thoracolumbar curve (Table 5).

**Table 5:** Association between incorrect posture and thoracolumbar curve among adolescents.

Variable	Univariate LR			Multivariate LR		
	OR	95% CI	*p*	OR	95% CI	*p*
Gender						
Boys	1.000			1.000		
Girls	1.936	1.754–2.138	**< 0.001**	1.680	1.511–1.869	**< 0.001**
Age (year)						
< 10	1.000			1.000		
10–15	2.054	1.820–2.319	**< 0.001**	1.665	1.405–1.974	**< 0.001**
> 15	1.863	1.563–2.222	**< 0.001**	1.681	1.272–2.222	**< 0.001**
School category						
Primary school	1.000			1.000		
Junior high school	1.426	1.295–1.571	**< 0.001**	0.876	0.764–1.005	0.058
Senior high school	1.320	1.163–1.498	**< 0.001**	0.834	0.669–1.039	0.106
Risser sign						
0	1.000			1.000		
1	1.749	1.426–2.146	**< 0.001**	1.215	0.969–1.525	0.092
2	1.646	1.383–1.959	**< 0.001**	1.143	0.933–1.400	0.197
3	1.744	1.523–1.997	**< 0.001**	1.225	1.026–1.463	**0.025**
4	2.003	1.785–2.248	**< 0.001**	1.468	1.232–1.750	**< 0.001**
5	1.627	1.370–1.931	**< 0.001**	1.334	1.037–1.715	**0.025**
Coronal plane						
Shoulder-height difference						
Normal	1.000			1.000		
Left height	1.039	0.901–1.198	0.599	0.796	0.589–1.076	0.138
Right height	1.215	1.055–1.399	**0.007**	0.704	0.525–0.944	**0.019**
Scapula tilt						
Normal	1.000			1.000		
Left tilt	1.147	0.996–1.321	0.058	1.193	0.885–1.609	0.247
Right tilt	1.369	1.191–1.575	**< 0.001**	1.436	1.072–1.924	**0.015**
Lumbar concave						
Normal	1.000			1.000		
Left concave	1.233	1.100–1.382	**< 0.001**	1.042	0.919–1.181	0.522
Right concave	1.440	1.300–1.595	**< 0.001**	1.137	1.013–1.277	**0.029**
Pelvic tilt						
Normal	1.000			1.000		
Left tilt	0.893	0.767–1.041	0.148	0.877	0.744–1.035	0.121
Right tilt	1.662	1.496–1.845	**< 0.001**	1.411	1.253–1.588	**< 0.001**
Sagittal plane						
Flat back						
Normal	1.000			1.000		
Abnormal	0.952	0.479–1.892	0.889	0.710	0.353–1.429	0.337
Hump back						
Normal	1.000			1.000		
Abnormal	0.730	0.460–1.159	0.182	0.853	0.528–1.377	0.515
Adam’s test						
Rib hump						
Normal	1.000			1.000		
Left eminence	0.684	0.589–0.795	**< 0.001**	0.606	0.473–0.777	**< 0.001**
Right eminence	1.482	1.351–1.626	**< 0.001**	1.452	1.215–1.736	**< 0.001**
Angle of thoracic rotation						
Normal (ATR 0–4°)	1.000			1.000		
Left (ATR ≥ 5°)	0.672	0.563–0.802	**< 0.001**	1.175	0.875–1.579	0.284
Right (ATR ≥ 5°)	1.452	1.321–1.597	**< 0.001**	1.076	0.893–1.296	0.442
Thoracolumbar eminence						
Normal	1.000			1.000		
Left eminence	0.606	0.478–0.768	**< 0.001**	0.986	0.525–1.853	0.966
Right eminence	0.760	0.636–0.909	**0.003**	1.409	0.870–2.283	0.163
Angle of thoracolumbar rotation						
Normal (ATR 0–4°)	1.000			1.000		
Left (ATR ≥ 5°)	0.592	0.459–0.765	**< 0.001**	0.746	0.379–1.468	0.396
Right (ATR ≥ 5°)	0.740	0.611–0.896	**0.002**	0.734	0.439–1.226	0.238
Lumbar eminence						
Normal	1.000			1.000		
Left eminence	1.630	1.481–1.793	**< 0.001**	1.762	1.486–2.089	**< 0.001**
Right eminence	0.990	0.868–1.130	0.883	1.067	0.829–1.372	0.616
Angle of lumbar rotation						
Normal (ATR 0–4°)	1.000			1.000		
Left (ATR ≥ 5°)	1.444	1.314–1.586	**< 0.001**	0.954	0.806–1.129	0.583
Right (ATR ≥ 5°)	0.884	0.768–1.017	0.084	1.013	0.770–1.331	0.929

OR, odds ratio; CI, confidence interval; ATR, angle of trunk rotation. The bold numbers of *p* value represent the significant differences.

#### 3.4.3 Analysis of related factors for the lumbar curve

Univariate LR analysis models were used to explore the factors associated with the lumbar curve. We found that female sex, age, Risser sign, lumbar concavity, pelvic tilt, left thoracolumbar eminence, left thoracolumbar ATR ≥ 5°, left lumbar eminence, and lumbar ATR ≥ 5° were significantly associated with the lumbar curve (*p* < 0.05). However, the right rib hump (OR: 0.853, 95% CI: 0.775–0.939, *p* = 0.001) and right thoracic ATR ≥ 5° (OR: 0.793, 95% CI: 0.716–0.877, *p* < 0.001) were negatively associated with the lumbar curve. In the multi-factor model, female sex (OR: 1.611, 95% CI: 1.453–1.787, *p* < 0.001), right lumbar concavity (OR: 1.371, 95% CI: 1.223–1.535, *p* < 0.001), left pelvic tilt (OR: 1.266, 95% CI: 1.085–1.477, *p* = 0.003), right pelvic tilt (OR: 1.321, 95% CI: 1.175–1.485, *p* = 0.003), left lumbar eminence (OR: 1.229, 95% CI: 1.033–1.462, *p* = 0.020), and right lumbar ATR ≥ 5° (OR: 1.360, 95% CI: 1.036–1.786, *p* = 0.027) were still associated with the lumbar curve. Interestingly, right shoulder height (OR: 0.741, 95% CI: 0.557–0.986, *p* = 0.039) was newly identified as negatively associated with the lumbar curve, and right thoracic ATR ≥ 5° (OR: 0.758, 95% CI: 0.627–0.917, *p* = 0.004) remained negatively associated with the lumbar curve. These results suggest that female sex, right lumbar concavity, pelvic tilt, left lumbar eminence, and right lumbar ATR ≥ 5° are independently associated factors for the lumbar curve, whereas the right shoulder height and right thoracic ATR ≥ 5° are negatively associated (Table 6).

**Table 6:** Association between incorrect posture and lumbar curve among adolescents.

Variable	Univariate LR			Multivariate LR		
	OR	95% CI	*p*	OR	95% CI	*p*
Gender						
Boys	1.000			1.000		
Girls	1.639	1.487–1.806	**< 0.001**	1.611	1.453–1.787	**< 0.001**
Age (year)						
< 10	1.000			1.000		
10–15	1.244	1.114–1.390	**< 0.001**	1.092	0.929–1.284	0.287
> 15	1.113	0.937–1.322	0.222	1.101	0.832–1.456	0.501
School category						
Primary school	1.000			1.000		
Junior high school	1.162	1.056–1.278	**0.002**	1.068	0.930–1.225	0.353
Senior high school	0.999	0.878–1.136	0.983	0.927	0.739–1.163	0.514
Risser sign						
0	1.000			1.000		
1	1.378	1.127–1.685	**0.002**	1.131	0.905–1.412	0.278
2	1.342	1.133–1.591	**0.001**	1.157	0.949–1.411	0.150
3	1.294	1.134–1.477	**< 0.001**	1.100	0.923–1.310	0.287
4	1.205	1.076–1.349	**0.001**	1.062	0.892–1.264	0.498
5	1.203	1.015–1.425	0.033	1.204	0.935–1.549	0.150
Coronal plane						
Shoulder-height difference						
Normal	1.000			1.000		
Left height	0.940	0.819–1.080	0.383	0.965	0.722–1.289	0.807
Right height	0.918	0.800–1.053	0.221	0.741	0.557–0.986	**0.039**
Scapula tilt						
Normal	1.000			1.000		
Left tilt	0.970	0.847–1.110	0.656	0.905	0.680–1.205	0.496
Right tilt	0.969	0.846–1.110	0.651	1.240	0.934–1.646	0.136
Lumbar concave						
Normal	1.000			1.000		
Left concave	1.160	1.033–1.304	**0.012**	1.009	0.889–1.146	0.884
Right concave	1.612	1.455–1.786	**< 0.001**	1.371	1.223–1.535	**< 0.001**
Pelvic tilt						
Normal	1.000			1.000		
Left tilt	1.289	1.117–1.487	**0.001**	1.266	1.085–1.477	**0.003**
Right tilt	1.626	1.462–1.808	**< 0.001**	1.321	1.175–1.485	**< 0.001**
Sagittal plane						
Flat back						
Normal	1.000			1.000		
Abnormal	1.678	0.903–3.119	0.102	1.740	0.925–3.275	0.086
Hump back						
Normal	1.000			1.000		
Abnormal	1.110	0.731–1.685	0.625	1.279	0.833–1.963	0.260
Adam’s test						
Rib hump						
Normal	1.000			1.000		
Left eminence	0.916	0.800–1.047	0.199	0.949	0.761–1.183	0.639
Right eminence	0.853	0.775–0.939	**0.001**	1.140	0.954–1.363	0.150
Angle of thoracic rotation						
Normal (ATR 0–4°)	1.000			1.000		
Left (ATR ≥ 5°)	0.908	0.776–1.064	0.233	1.082	0.834–1.404	0.552
Right (ATR ≥ 5°)	0.793	0.716–0.877	**< 0.001**	0.758	0.627–0.917	**0.004**
Thoracolumbar eminence						
Normal	1.000			1.000		
Left eminence	1.821	1.503–2.205	**< 0.001**	1.276	0.726–2.243	0.396
Right eminence	1.107	0.937–1.309	0.232	1.335	0.833–2.138	0.230
Angle of thoracolumbar rotation						
Normal (ATR 0–4°)	1.000			1.000		
Left (ATR ≥ 5°)	1.928	1.572–2.363	**< 0.001**	1.465	0.808–2.657	0.208
Right (ATR ≥ 5°)	1.109	0.929–1.325	0.253	0.955	0.581–1.570	0.857
Lumbar eminence						
Normal	1.000			1.000		
Left eminence	1.249	1.134–1.375	**< 0.001**	1.229	1.033–1.462	**0.020**
Right eminence	1.159	1.022–1.315	**0.021**	0.992	0.767–1.281	0.949
Angle of lumbar rotation						
Normal (ATR 0–4°)	1.000			1.000		
Left (ATR ≥ 5°)	1.230	1.117–1.353	**< 0.001**	0.943	0.794–1.121	0.509
Right (ATR ≥ 5°)	1.227	1.076–1.400	**0.002**	1.360	1.036–1.786	**0.027**

OR, odds ratio; CI, confidence interval; ATR, angle of trunk rotation. The bold numbers of *p* value represent the significant differences.

## 4 Discussion

Back signs play a crucial role in the early detection and diagnosis of AIS (21, 22). Our research team previously studied the correlation between incorrect posture and the curve magnitude in patients with AIS (19). However, the relationship between incorrect posture and different curve types remains unclear. To the best of our knowledge, this is the first large-scale cross-sectional study to investigate the relationships between sex, age, Risser sign, incorrect posture, trunk rotation angle, and curve type in patients with AIS. Our results showed that pelvic tilt, ATR, and right rib hump may serve as the most reliable indicators of curve types in AIS.

Incorrect posture is significantly associated with the Cobb angle. This study found a considerably higher proportion of female patients with spinal curves across various bending amplitudes. The highest prevalence was observed among patients aged between 11 and 15 years. Adolescence is a critical period for the development of spinal curvature, and the accelerated growth rate and changes in hormone levels during this period may contribute to the increased incidence of AIS in females (23, 24). In addition, estrogen may play an important role in the development of the spinal curve (25). We found that shoulder height difference, scapular tilt, lumbar concavity, pelvic tilt, rib hump, and ATR were significantly and positively correlated with the Cobb angle. Specifically, when the Cobb angle was larger, the proportion of patients with right rib hump and right thoracic ATR ≥ 5° was higher. Our previous study identified the right rib hump as the best indicator of spinal curve severity (14). Supporting this, Ferraro et al. (26) discovered that, for every 1-mm increase in the height of the rib protrusion—as measured using the humpmeter—the Cobb angle increased by an average of 1.542° to 1.857°. This indicates that the rib hump is a reliable indicator of spinal curve severity. Therefore, the right rib hump can not only predict the curve magnitude of AIS but also the curve type and, thus, should receive special attention in school-based scoliosis screenings.

Incorrect posture is associated with the curve type. During an in-depth analysis of the factors influencing the curve type, we found a significant correlation between curve types and specific incorrect postures. Moreover, female sex was an independent associated factor for all the curve types. Around the peak of the growth spurt, mid-thoracic levels in girls are less mature than those in boys, potentially influencing the development of AIS (27). In the thoracic curve group, the incidence of incorrect posture on the right side (including right shoulder height, right scapular tilt, and right rib hump) was significantly higher than that on the left side. By contrast, in the lumbar curve group, the primary manifestations were left lumbar eminence and right lumbar concavity, which may be related to an imbalance in neuromuscular control or differences in the biomechanical load. Studies have shown that patients with a thoracic curve have poor coordination between the movement of the lumbar spine and the thorax when performing trunk rotation, which may lead to the development of an abnormal posture (28). Moreover, the characteristics of the thoracic curve and wedge-shaped deformation of the intervertebral discs demonstrate significant differences across the spinal stiffness groups, which may also affect the stability and control of the posture (29).

Right-sided postural abnormalities appear to be closely associated with the thoracic curve. Interestingly, our results revealed that right-sided postural abnormalities such as the right scapular tilt, right pelvic tilt, right rib hump, and right thoracic ATR ≥ 5° are associated factors for the thoracic curve. This result is similar to that of Sapkas et al. (30), who observed a statistically significant correlation between the thoracic ATR and the thoracic Cobb angle. Notably, the incidence of the right thoracic curve has been reported to range between 80 and 99% (31). A previous study indicated that the thoracic organ orientation may influence the direction of convexity in AIS (32). Although the etiology of the direction of scoliosis remains unclear, this could explain the reason for a strong association between a right-sided incorrect posture and the thoracic curve than that between a left-sided incorrect posture and the thoracic curve in this study. This study also showed that a right-sided incorrect posture is associated with the thoracolumbar curve. Although right thoracic ATR ≥ 5° was identified as an associated factor for the thoracic curve, the right shoulder height and right thoracic ATR ≥ 5° were negatively associated with the lumbar curve. Therefore, in school-based scoliosis screenings, special attention should be paid to students with a thoracic ATR of ≥ 5°.

Pelvic tilt is closely associated with the curve type in AIS. The results showed that the right pelvic tilt not only increased the risk of thoracic and thoracolumbar curves but also showed a significant association between the lumbar curve and bilateral pelvic inclination. Dalleau et al. (15) indicated that pelvic tilt may affect the overall balance and postural stability of the spine, leading to compensatory curves. Pelvic tilt may also alter the position of the acetabulum and change the force line of the lower limbs, thereby affecting the overall balance of the spine (33). This indicates that the symmetry and mechanical balance of the pelvis are crucial for maintaining the normal curve of the spine.

While our ROC analysis showed that a right thoracic ATR ≥ 5° was the strongest single predictor for a thoracic curve (AUC 0.629), the discriminative performance of ATR alone was modest, with other ATR measures yielding AUCs near 0.5. This indicates that although postural signs and ATR are significantly associated with curve type, they should not be used as standalone classifiers. Instead, they are best employed in combination with the forward bend test, clinical judgment, and other physical signs (e.g., rib hump, pelvic tilt) within a multi-step screening pathway. This practical approach aligns with how these indicators are used in real-world school screening programs.

Since recent years, several notable advancements have emerged in the field of AIS assessment and management. Recent systematic evidence has reaffirmed that school-based screening programs, when implemented with well-designed protocols, remain both cost-effective and clinically valuable for early detection (34). The validation of home-use screening tools and AI-powered monitoring applications points toward a future model of decentralized, patient-engaged surveillance (35, 36). These innovations have the potential to complement traditional screening approaches by reducing healthcare system burdens while preserving diagnostic accuracy. Our findings on the association between specific incorrect postures and distinct curve types have important clinical implications. Accumulating evidence supporting the Schroth method underscores the importance of integrating physiotherapeutic scoliosis-specific exercises into conservative management protocols (37). Given the heterogeneity of curve patterns and severity in AIS, selecting individualized treatment strategies based on curve type, magnitude, and patient-specific characteristics is essential for optimizing outcomes (38, 39). The identification of curve type-specific postural indicators, such as right rib hump for thoracic curves and left lumbar eminence for lumbar curves, may facilitate more targeted and personalized exercise prescription.

This study has several limitations. First, this was a cross-sectional study based on large-scale data, making it difficult to make causal inferences between the associated factors and the spinal curve. Second, some participants may have had more than one spinal curve, but we analyzed only the influencing factors of a single curve type. In future studies, we aim to combine different curve types to analyze their influencing factors. Third, although our postural assessments followed with two independent therapists and a senior therapist, formal inter-rater reliability statistics (e.g., kappa or ICC) were not calculated for this large-scale screening. Finally, owing to the large scale of screening, some potential influencing factors associated with AIS were not adequately examined in this study, including BMI, physical activity, family history, genetic factors, hormone levels, and nutritional status. We plan to incorporate these factors into our subsequent studies to further explore their relationships with curve patterns.

## 5 Conclusion

Despite these limitations, our study—based on a large sample size (*n* = 10,453)—is among the first to demonstrate a significant association between incorrect posture and both the curve magnitude and curve type. Notably, right-sided features such as right rib hump exhibited a predictive value for both the curve magnitude and thoracic curve, whereas pelvic tilt and lumbar eminence emerged as key physical signs related to thoracolumbar and lumbar curves. Additionally, a right thoracic ATR of ≥ 5° is not only associated with the thoracic curve but also is negatively associated with the lumbar curve. Therefore, this study suggests that the Cobb angle magnitude and curve type are meaningfully associated with postural signs. Early detection of curve patterns using postural indicators may facilitate timely intervention, enabling early prevention and early treatment strategies that could improve clinical outcomes in AIS.

## Data Availability

The original contributions presented in the study are included in the article/Supplementary material, further inquiries can be directed to the corresponding author/s.
