# Supplementary material for: Association between incorrect posture and curve types in adolescent idiopathic scoliosis: a large-sample, cross-sectional study in China
Source: Front Public Health. 2026 Jun 12;14:1785027. doi: 10.3389/fpubh.2026.1785027 (PMC13303964; doi:10.3389/fpubh.2026.1785027)

Figure S1. ROC Curve Analysis of ATR for Thoracic Curve

ROC curve results of left and right thoracic rotation angle, thoracolumbar rotation angle, and lumbar rotation angle for thoracic curve.

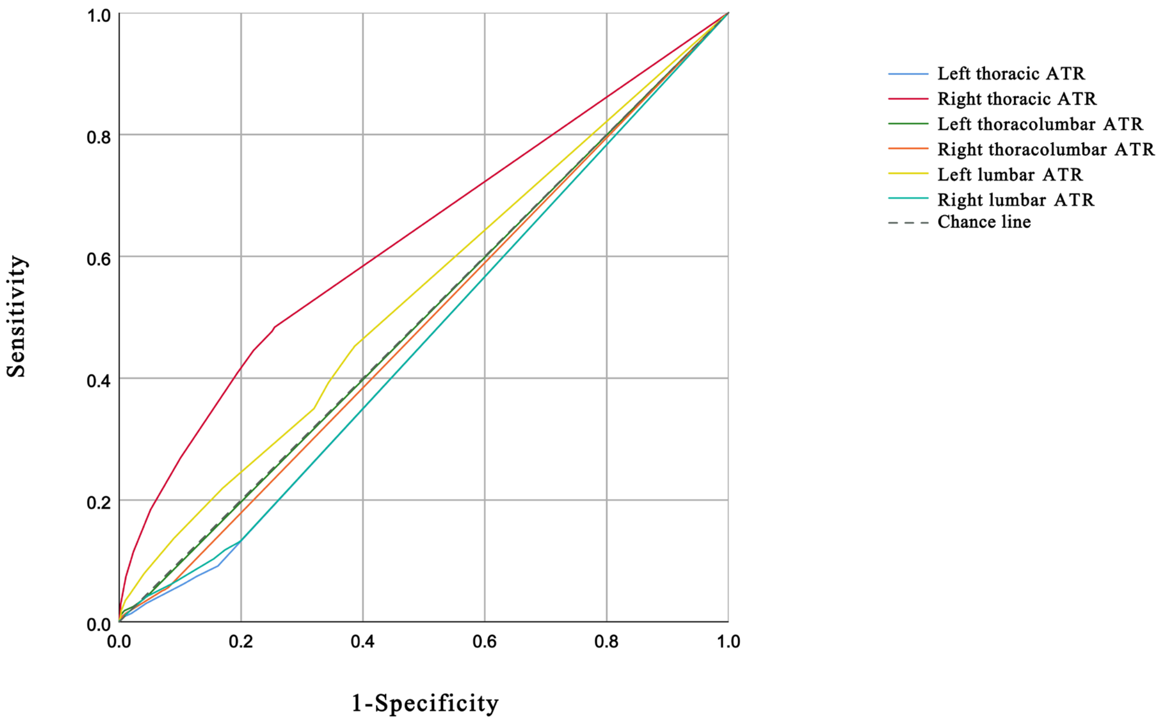

Supplement: Supplementary file 1 [file Image_1.pdf]
