# Supplementary material for: Association between incorrect posture and curve types in adolescent idiopathic scoliosis: a large-sample, cross-sectional study in China
Source: Front Public Health. 2026 Jun 12;14:1785027. doi: 10.3389/fpubh.2026.1785027 (PMC13303964; doi:10.3389/fpubh.2026.1785027)
Supplement: Supplementary file 2 [file Supplementary_file_1.docx]

All physical sign assessments in this study were conducted in strict accordance with the *Chinese National Standard (GB/T 16133-2014)* and combined with clinical practice guidelines for adolescent idiopathic scoliosis (AIS) screening and diagnosis. The operational definitions of each physical sign involved in the study are standardized and clarified as follows:

**Shoulder-height difference:** Assessed in the upright standing position (feet shoulder-width apart, arms naturally hanging down, head in neutral position). The vertical distance between the acromion processes of the two sides was measured with a height gauge, and divided into left shoulder height (left acromion higher than right) and right shoulder height (right acromion higher than left).

**Scapula tilt:** Evaluated by the position of the inferior angle of the scapula in the upright position. The line connecting the inferior angles of the two scapulae was compared with the horizontal line, including left tilt (left inferior angle lower than right) and right tilt (right inferior angle lower than left).

**Lumbar concave:** Observed in the upright position, with the trunk in a neutral state. The degree of concavity of the paravertebral soft tissue on both sides of the lumbar spine was assessed; an obvious asymmetric concavity (one side significantly more concave than the other) was defined as abnormal, divided into left lumbar concave and right lumbar concave.

**Pelvic tilt:** Measured in the upright position using a pelvic inclinometer at the anterior superior iliac spine (ASIS). A difference in the vertical height of the bilateral ASIS ≥3 mm or a pelvic obliquity angle ≥3° was defined as pelvic tilt, including left tilt (left ASIS lower than right) and right tilt (right ASIS lower than left).

**Rib hump:** Assessed with the patient in forward bending position (knees straight, trunk bent forward 90° from the hips, arms hanging down naturally). A palpable or visible asymmetric protrusion of the rib cage on either side was defined as a rib hump; the side with the obvious protrusion was recorded as left/right rib hump.

**Lumbar eminence:** In the forward bending position, an asymmetric protrusion of the paravertebral muscle or bony structure of the lumbar spine was defined as lumbar eminence; the side with the obvious protrusion was recorded as left/right lumbar eminence.

**Thoracolumbar eminence:** In the forward bending position, an asymmetric protrusion at the thoracolumbar junction was defined as thoracolumbar eminence; the side with the obvious protrusion was recorded as left/right thoracolumbar eminence.

**Angle of trunk rotation (ATR):** Measured at the corresponding spinal segments (thoracic, thoracolumbar, lumbar) in the forward bending position using a standard scoliometer. The measurement was performed three times, and the average value was taken; ATR ≥5° was defined as abnormal trunk rotation, and divided into left/right rotation according to the direction of the scoliometer’s indication.
