# Supplementary material for: Association between incorrect posture and curve types in adolescent idiopathic scoliosis: a large-sample, cross-sectional study in China
Source: Front Public Health. 2026 Jun 12;14:1785027. doi: 10.3389/fpubh.2026.1785027 (PMC13303964; doi:10.3389/fpubh.2026.1785027)
Supplement: Supplementary file 3 [file Table_1.DOCX]

**Table S1. AUC scores of ATR for Thoracic Curve**

| **Predictor Variables** | Area | Std. Error¹ | Asymptotic Sig.² | **Asymptotic 95% CI** | |
| --- | --- | --- | --- | --- | --- |
|  |  |  |  | Lower Bound | Upper Bound |
| Left thoracic ATR | 0.465 | 0.006 | 0.000 | 0.454 | 0.476 |
| Rigt thoracic ATR | 0.629 | 0.006 | 0.000 | 0.618 | 0.640 |
| Left thoracolumbar ATR | 0.498 | 0.006 | 0.767 | 0.487 | 0.510 |
| Right thoracolumbar ATR | 0.488 | 0.006 | 0.039 | 0.477 | 0.499 |
| Left lumbar ATR | 0.537 | 0.006 | 0.000 | 0.526 | 0.548 |
| Right lumbar ATR | 0.468 | 0.006 | 0.000 | 0.457 | 0.479 |

¹ Standard Error

² Asymptotic Significance
